# The complete mitochondrial genome of *Atypus karschi* (Araneae, Atypidae) with phylogenetic consideration

**DOI:** 10.1080/23802359.2021.1959443

**Published:** 2021-07-28

**Authors:** Ailan He, Jing Guo, Hongyuan Peng, Zongguang Huang, Jinxin Liu, Xiang Xu

**Affiliations:** a Henry Fok School of Biology and Agriculture, Shaoguan University, Shaoguan, China; b College of Life Science, Hunan Normal University, Changsha, China

**Keywords:** *Atypus karschi*, mitochondrial genome, NGS technique, Atypidae

## Abstract

The complete mitochondrial genome sequence of *Atypus karschi* has a circular genome of 14,149 bp, comprised of 13 protein-coding genes, two rRNA genes, 22 tRNA genes, and a control region. The nucleotide composition is 35.82% of T, 35.13% of A, 17.19% of G, and 9.16% of C. Most genes are encoded on the heavy strand except seven tRNA genes (*Leu*, *Phe*, *His*, *Pro*, *Leu*, *Ile*, *Gln*), four protein-coding genes (*nad5*, *nad4*, *nad4l*, *nad1*), and 16S-rRNA on the light strand. Most protein-coding genes start with TTG, ATT or ATA initiation codon except cox1, cox1’s start codon cannot be determined, and three types of inferred termination codons are TAA, TAG, and an incomplete stop codon. There are four intergenic spacers and 25 gene overlaps. The phylogenetic analysis shows that *A. karschi* has closer genetic relationship with *Cyriopagopus schmidti* (von Wirth, 1991) and *Phyxioschema suthepium* (Raven & Schwendinger, 1898) with high bootstrap support.

*Atypus karschi* (Dönitz, 1887), the purse-web spider is distributed in China, Korea and Japan. In China, it is a common species but only in the south can be found (Zhu et al. 2006; Yin et al. 2012; WSC 2020). Due to the destruction of their natural habitats and decreasing populations, *A. karschi* was listed as a near threatened species by China Species Red List (Wang and Xie 2004).

So far there are only a few researches on the life cycle and morphology of *A. karschi* (Yin et al. 1983; Feng 1990 ; Song et al. 1999; Zhu et al. 2006; Yin et al. 2012; WSC 2020), and no any mitochondrial genomes of the genus *Atypus* in NCBI PubMed. We sequenced the complete mitochondrial genome of *A. karschi* using the next-generation sequencing (NGS) techniques for its further population genetics and polymorphism studies. The specimen was collected from Mangshan National Nature Reserve (24°57'N, 112°58'E), Chenzhou City, Hunan Province, China. The muscle of legs of *A. karschi* (voucher no. M1204) was preserved in 95% ethanol and stored at −20 °C refrigerator in Hunan Normal University, Changsha, China (contact person: Xiang Xu, email: xux@hunnu.edu.cn). The procedure referred from Green and Sambrook (2012) was carried out in the total genomic DNA extraction.

We sequenced and characterized the complete mitochondrial genome of *A. karschi* in our study. The genomic DNA was extracted by using a Universal Genomic DNA Kit (CoWin Biosciences, China), and DNA sample quality and quantity were characterized by gel electrophoresis and Nano-Drop 2000 spectrometer (ThermoFisher Scientific, USA). The high-quality genomic DNA was used to prepare DNA library, with insert sizes of 350 bp for paired-end sequencing. All data were generated on an Illumina Novaseq6000 using sequencing protocols provided by the manufacturer (Illumina, USA). Approximately 4.13 G raw data were generated with read lengths of 150 bp. Illumina paired-end reads were filtered based on quality values, and the low-quality reads were trimmed. The remaining clean reads were used for assembly with SPAdes v.3.5.0 (http://cab.spbu.ru/software/spades/) based on overlapping and paired-end relationships. The mitogenome was annotated using MITOS webserver (Bernt et al. 2013). The annotations were rechecked by BLAST search in the Nt and Nr databases (https://www.ncbi.nlm.nih.gov). The mitochondrial DNA sequence of *A. karschi* with the annotated genes was deposited in GenBank (accession number: MT832081). The complete mitochondrial genome sequence of *A. karschi* is a circular one of 14,149 bp and contains 13 protein-coding genes, two ribosomal RNA genes (12S-rRNA and 16S-rRNA), 22 transfer RNA genes (tRNA), and a control region. Most of them are encoded on the heavy strand except seven tRNA genes (*Leu*, *Phe*, *His*, *Pro*, *Leu*, *Ile*, *Gln*), four protein-coding genes (*nad5*, *nad4*, *nad4l*, *nad1*), and 16S-rRNA. The GC percent of 37 genes varies from 12.50% to 36.00%. The overall nucleotide composition in descending order is 35.82% of T, 35.13% of A, 17.19% of G and 9.16% of C. The most representative base is T, and the bias against C is observed. Among 13 protein-coding genes (total 10,705 bp) encoding 3556 amino acids, the maximum is *nad5* with 1644 bp and the minimum is *atp8* with only 156 bp. The 12S-rRNA and 16S-rRNA genes are 636 and 746 bp, respectively located between the tRNA^Ile^ and tRNA^Leu^ genes and separated by the tRNA^Val^ and tRNA^Tyr^ genes.

Most protein-coding genes start with TTG (*cox2*, *cox3*, *nad4*, *nad6*, *cob*) or ATT (*atp8*, *nad5*, *nad4l*, *nad1*) or ATA (*nad2*, *atp6*, *nad3*) initiation codon except *cox1*, and three types of inferred termination codons are TAA (*cox1*, *cox2*, *atp8*, *atp6*, *cox3*, *nad5*, *nad4l*, *nad6*, *cob*, *nad1*), TAG (*nad2*, *nad3*) and an incomplete stop codon (*nad4*). 22 tRNA genes vary from 46 to 74 bp in length, and 18 of them fold into the typical cloverleaf secondary structure. There are four intergenic spacers (total 203 bp) varying from 1 to 195 bp in length and 25 gene overlaps (total 249 bp), the largest of which is 33 bp between the tRNA^Arg^ and tRNA^phe^ genes. The tandem repeat sequences are observed in tRNA^Phe^ (GAA), nad5 and control region.

We constructed the phylogenetic relationship of 25 Araneae species based on molecular data of complete mitochondrial genomes to test the placement of *A*. *karschi* in phylogeny of the order Araneae. We analyzed under maximum-likehood (ML), and used RAxML (version 8, Stamatakis 2014). The best-fit model GTR + G+ I was selected using JModeltest program under the Akaike information criterion (AIC). The phylogenetic tree (Figure 1) shows the clade (*A. karschi* (*Calisoga longitarsis* (*Phyxioschema suthepium* (*Haplopelma schmidti*, *Cyriopagopus hainanus*) has been supported strongly by bootstrap value. Members of this clade have a closer genetic relationship than other species.

**Figure 1.**
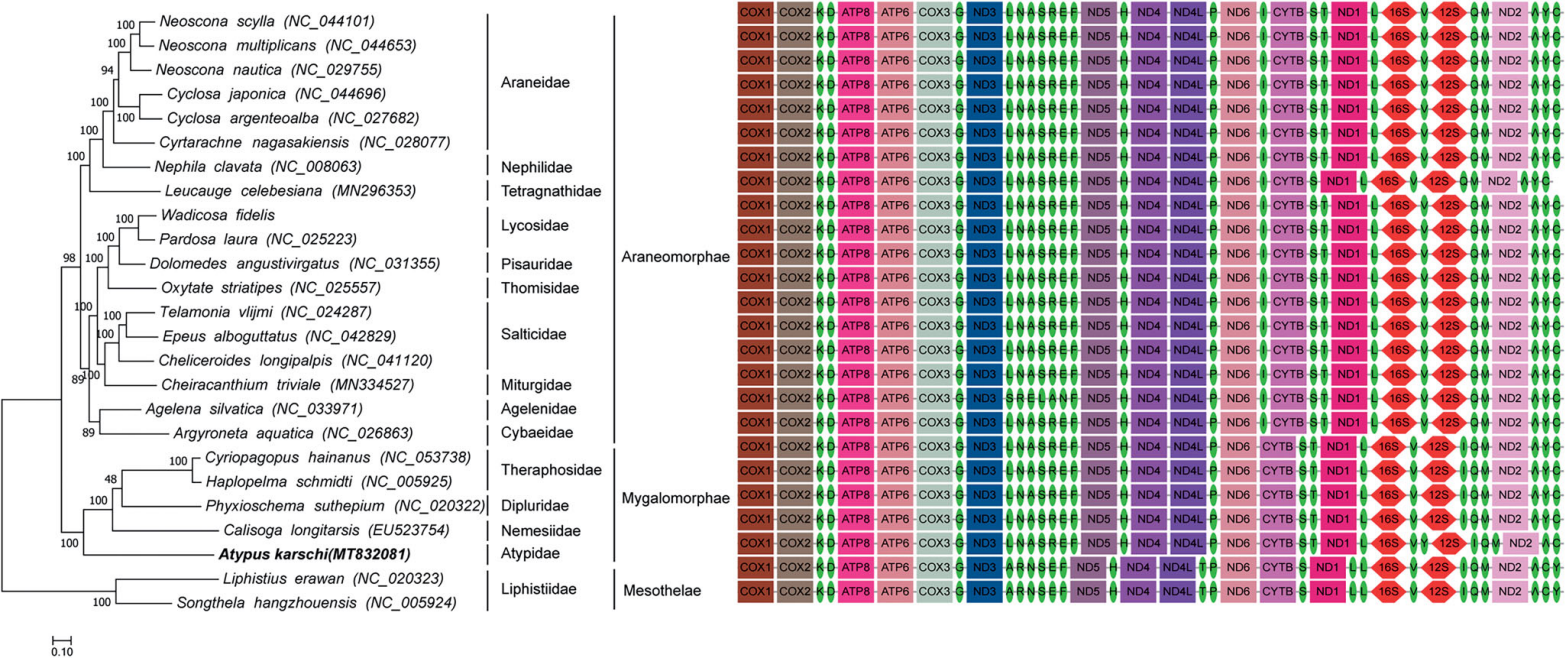
Phylogenetic tree of maximum-likelihood was constructed in RAxML based on the nucleotide sequences of 13 protein-coding genes. The rectangle, ellipse and horizontal hexagon in gene order represent coding gene, tRNA and rRNA, respectively.

This result is consistent with the result of morphological taxonomy on Order Araneae. Order Araneae is divided into two suborders, Mesothelae and Opisthothelae. *Songthela hangzhouensis* and *Liphistius erawan* belong to the family Liphisiidae, the only family in the Suborder Mesothelae. They are the spiders having a segmented series of plates on the upper surface of their abdomens, and their spinnerets are situated on the middle part between the beginning and ending of ventral abdomens. The members of Suborder Opisthothelae have no segmented plates and their spinnerets are situated at or near the ending of ventral abdomens, which are completely different from those of Mesothelae. Therefore, suborder Opisthothelae is divided into two infraorders, Mygolomorphae and Araneomorphae. In the present study, *A. karschi* and its sister lineage, including *Cyriopagopus schmidti* (Theraphosidae), *Haplopelma schmidti* (Theraphosidae), *Phyxioschema suthepium* (Euagridae) and *Calisoga longitarsis* (Nemesiidae), belong to the Infraorder Mygolomorphae, and the other 18 species belong to the Infraorder Araneomorphae.

## Data Availability

The genome sequence data that support the findings of this study are openly available in GenBank of NCBI at https://www.ncbi.nlm.nih.gov/ under the accession no. MT832081. The associated BioProject, SRA, and Bio-Sample numbers are PRJNA741850, SRR14935285, and SAMN19909606, respectively.
